# Association between poverty, low educational level and smoking with adolescent’s working memory: cross lagged analysis from longitudinal data

**DOI:** 10.3389/fpubh.2024.1341501

**Published:** 2024-03-25

**Authors:** Hari Wahyu Nugroho, Harsono Salimo, Hartono Hartono, Moh Abdul Hakim, Ari Probandari

**Affiliations:** ^1^Doctoral Program of Public Health, Faculty of Medicine, Universitas Sebelas Maret, Surakarta, Indonesia; ^2^Department of Public Health, Faculty of Medicine, Universitas Sebelas Maret, Surakarta, Indonesia

**Keywords:** working memory, social determinants, educational level, smoking, poverty

## Abstract

**Background:**

Working memory is a crucial element of cognitive function. Previous cross-sectional studies have identified various determinants of working memory in children and adolescents. Nonetheless, no study has yet demonstrated the causal relationship of social determinants with working memory in adolescents.

**Objective:**

This study explores the causal link between the level of education, smoking, and other factors with adolescent’s working memory.

**Methods:**

This study analyzed secondary data from waves 4 and 5 of the Indonesia Family Life Survey (IFLS), utilizing cross-lagged analysis in Jamovi version 2.4.8. The variables included working memory score, maternal education, household income, stress, educational level, smoking, urbanicity, and physical activity. These variables were extracted from IFLS waves 4 and 5, and each dependent variable in IFLS wave four was controlled by the same dependent variables in IFLS 5. Then, we used cross-lagged analysis to assess the causality between each dependent variable and a working memory score in IFLS wave 5.

**Result:**

The findings indicate that level of education had a positive impact on working memory in adolescents aged 15–18 years, with a Beta value of 0.18 (95% CI 0.81–0.2; *p* < 0.001). Smoking and age were negatively associated with working memory, with Beta values of −0.07 (95% CI -0.65 -0.04; *p* < 0.029) and − 0.10 (95% CI -0.25 -0.05; *p* < 0.003), respectively. No evidence was found for a significant correlation between poverty and adolescents’s working memory.

**Conclusion:**

The findings indicate that increased education levels are associated with improved working memory in adolescents aged 15–18. At the same time, smoking has a negative impact on working memory in this age group.

## 1 Introduction

### 1.1 Background

In developing nations, the specter of poverty continues to loom large, profoundly impacting the well-being and development of children. In response, the 2030 Agenda for Sustainable Development Goals (SDGs) places a marked emphasis on the mitigation of poverty, particularly within these countries. A notable aspect of this agenda, encapsulated in SDG 1, is the established correlation between enhanced nutrition and reduced poverty levels. The detrimental effects of poverty on the nutritional, health, and cognitive well-being of populations, especially children, have been extensively documented and recognized in various studies (1–3). Poverty, as a critical determinant of health, exerts a significant influence on the cognitive development of children (1).

Working memory begins to form from the age of 3–4 years, then reaches its peak capacity at the ages of 13–15 years. After that, the capacity of working memory will start to decline. The formation of working memory abilities starts with the development of self-inhibition and self-regulation, which each begin to form at the ages of 2 years and 4 years, respectively (4).

Memory is one crucial fundamental aspect of the construction of cognition. Memory, sensory, motor, inhibition control, processing speed, and attention are critical aspects for children in learning. Working memory is an essential part of memory (5).

Considering the importance of the role of working memory in cognitive function and school achievement, it is also crucial to understand the determinants related to working memory. In previous research, social determinants, including family income level, educational level, and place of residence, have been associated with child development and school achievement (6). A socioeconomic gap is related to language abilities, locomotor skills, cognitive abilities, spatial ability, and school readiness (7–9). Children living in poverty are suspected to experience a decrease in working memory capacity accompanied by an increase in allostatic load as one of the measures of physiological stress (10). Low family income levels can decrease the ability to provide essential play tools for stimulation, reducing a child’s working memory and cognitive abilities (11). Conversely, favorable socioeconomic conditions will increase and enrich stimulation and facilities, thus enhancing a child’s working memory capacity (12).

The impact of socioeconomic conditions on short-term memory remains a debatable subject. Several studies indicate no correlation between socioeconomic conditions and short-term memory (13–15). However, other research suggests otherwise, revealing that poor socioeconomic conditions can decrease working memory capacity (3, 16, 17).

The two most recent systematic reviews and meta-analyses, one of which was limited to developing countries, yielded comparable outcomes. Children exhibit impaired working memory due to socioeconomic disadvantage (18). Meanwhile, other systematic reviews and meta-analyses focusing on developing countries have revealed an association between poverty and low levels of education in mothers with reduced working memory in children (19). The studies included were conducted cross-sectionally to better understand and explore other factors’ impact on working memory. Various researchers suggest that future studies utilize longitudinal data and evaluate causality. The study specifically examines the relationship between educational level, smoking, and other factors and their effect on adolescents’s working memory.

### 1.2 Methods

This study entailed a secondary analysis of the 4th (2007) and 5th (2014) waves of the Indonesian Family Life Survey (IFLS), a longitudinal examination tracing the evolving socio-economic and health dynamics within Indonesian familial structures. Initiated in 1993, the IFLS initially encompassed a representative cohort from 13 out of 26 provinces, encapsulating approximately 83% of Indonesia’s demographic expanse. The survey’s scope comprehensively extends to individual and family profiles, household dynamics, community interactions, and the utilization of health and educational infrastructures. The inaugural IFLS survey in 1993 (IFLS1) engaged participants from 7,224 households. A subsequent iteration, IFLS2, revisited these respondents 4 years later. In the wake of Indonesia’s socio-economic turmoil in 1998, a specialized follow-up (IFLS2+) scrutinized the immediate repercussions on a 25% subset of the original sample. Ensuing surveys, IFLS3 in 2000, IFLS4 between late 2007 and early 2008, and IFLS5 spanning late 2014 to early 2015, consistently re-engaged the initial households and their offshoots. The latest wave, IFLS5, encompassed a substantial sample of 16,204 households and 50,148 individuals. IFLS conducts the sole survey that offers cross-sectional and longitudinal data in Indonesia. The Rand Corporation website provides publicly available data from the IFLS surveys.

We collected and analyzed data from two waves of datasets, including working memory, maternal education, household income, stress, inflammation, educational level, smoking, urban/non-urban dwelling, and physical activity. Our analysis resulted in a total of 894 adolescents, with complete matching data for each personal ID/PIDLINK number for IFLS waves 4 and 5.

#### 1.2.1 Working memory

The interviewer presented a list of 10 words, and participants were asked to recall as many words as they could within 2 minutes. A dummy variable was created to capture the number of correctly recalled words by each participant.

#### 1.2.2 Variables

Household income was assessed by calculating the ratio of expenditures on food and non-food items. We classified families as either poor or non-poor using a cut-off ratio of 20%. A family is classified as poor if their ratio of food to non-food expenditures exceeds 20%. Mothers’ and participants’ educational attainment was determined by their formal years of education. Stress levels were assessed utilizing a 10-question questionnaire. The questionnaire covered a range of topics including frequent disturbances from minor matters, challenges in maintaining focus, a sense of being overwhelmed by everyday responsibilities, experiences of fear or anxiety, sleep disturbances, feelings of isolation, and a decrease in motivation. Additionally, it inquired about the respondents’ sense of hope for the future and their overall level of happiness. Inflammation was assessed by C reactive protein (CRP) level with cutoff point 15 mg/L.

#### 1.2.3 Ethics

The IFLS survey and its procedures were approved by review boards in the US (at the Rand Corporation in Santa Monica, California) and in Indonesia (at Universitas Gadjah Mada in Yogyakarta and Universitas Indonesia in Jakarta). Every participant gave written informed consent to participate. For adolescents in the study, this consent was also obtained from their family members, caregivers, or legal guardians.

#### 1.2.4 Statistical analysis

**Figure 1:**
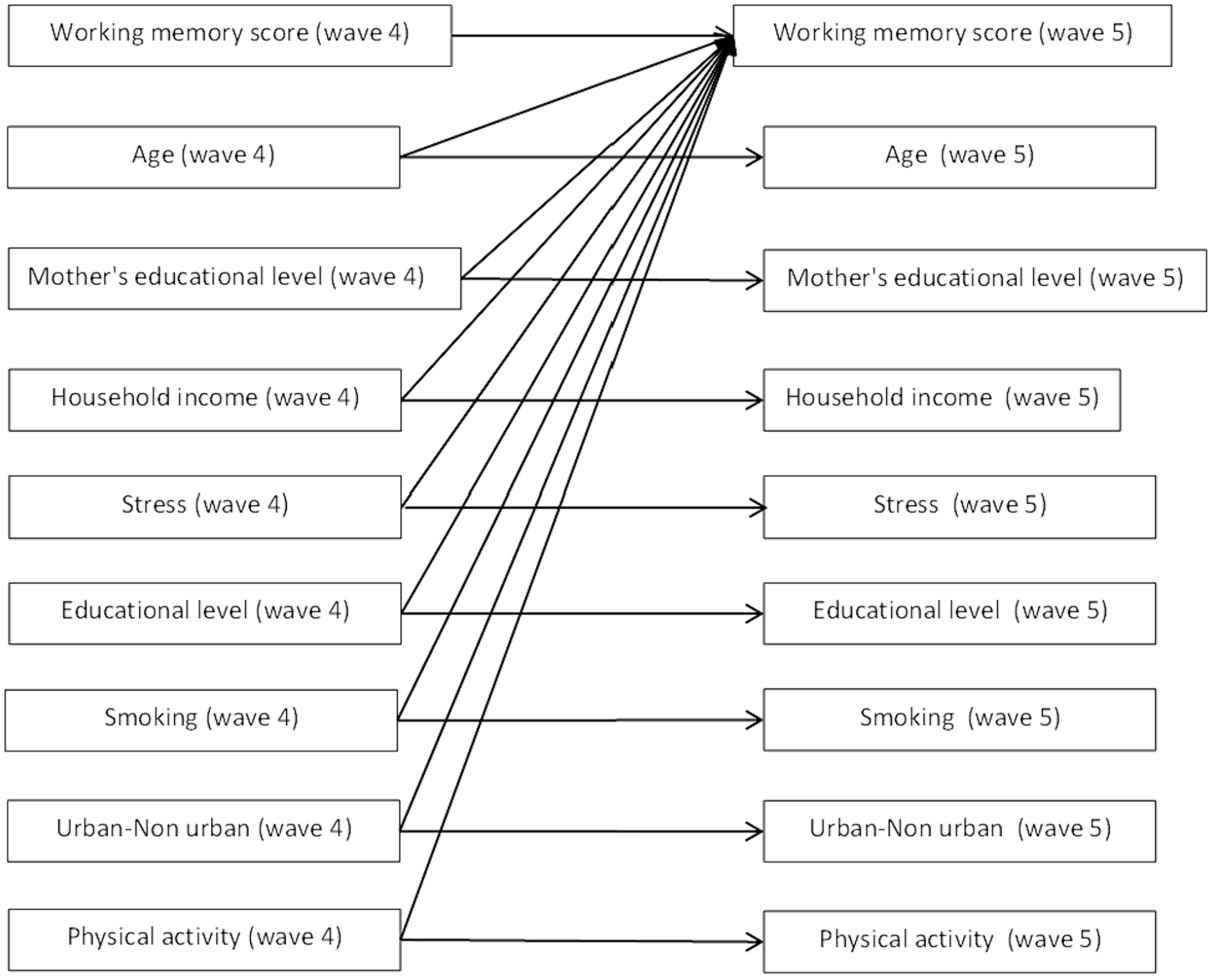
Cross lagged design.

The datasets extracted from IFLS were analyzed using Jamovi version 2.4.8. We performed a cross-lagged analysis in Jamovi. We set all variables in IFLS 5 as endogenous variables and all variables in IFLS 4 as exogenous covariates. Then, we set up a model in which exogenous covariates controlled each endogenous variable. We then analyzed the association between each variable and working memory score in IFLS 5 (Figure 1). In the parameter option, we use bootstrap with 10,000 bootstrap replications.

## 2 Results

### 2.1 Characteristic of demography

A total of 894 participants were identified from IFLS Wave 4 and IFLS Wave 5. Each participant shared the same PIDLINK, which ensured the same individual was followed. Demographic data indicates an increase in maternal and personal educational levels as well as household income from IFLS wave 4 (2007) to IFLS wave 5 (2014). Conversely, an increase in smoking prevalence among participants was observed (see Table 1).

**Table 1:** Sample demographic and clinical characteristics.

Variables	IFLS 4 (*n* = 894)		IFLS 5 (*n* = 894)	
	Mean ± SD	*n* (%)	Mean ± SD	*n* (%)
Sex				
Male		400 (44.74)		400 (44.74)
Female		494 (55.26)		494 (55.26)
Age	16.35 ± 1.04		23.68 ± 1.05	
Working memory	6.11 ± 2.39		5.92 ± 1.54	
Mother’s educational level (years)	5.93 ± 4.14		7.44 ± 4.39	
Educational level (years)	8.77 ± 1.99		11.16 ± 3.29	
Stress	1.49 ± 2.39		4.97 ± 1.99	
Household income				
Poor		357 (40.02)		289 (32.33)
Non-poor		535 (59.98)		605 (62.67)
Living				
Non-urban		360 (40.27)		449 (50.22)
Urban		534 (59.73)		445 (49.78)
Physical activity				
Yes		688 (77.74)		528 (59.06)
No		197 (22.26)		366 (40.94)
Smoking				
Yes		112 (12.66)		288 (32.21)
No		773 (87.34)		606 (67.79)

### 2.2 Outcome

Our study showed three variables significantly associated with working memory in adolescents aged 15–18. Specifically, a higher level of education had a positive association with working memory with a Beta score of 0.18 (95% CI 0.81–0.2; *p* < 0.001), while smoking had a negative association with working memory with a Beta score of −0.07 (95% CI −0.63−−0.03; *p* = 0.03). The data suggests that household income is not significantly associated with adolescents’s working memory, as indicated by a Beta score of 0.02 (95% CI -0.15 to 0.25; *p* < 0.7). A negative association with adolescents’s working memory was observed with age of −0.11 (95% CI -0.25 to −0.06; *p* < 0.002) (Table 2).

**Table 2:** Association between variables with adolescents’s working memory.

	95% Confidence intervals				
Variables	Lower	Upper	Β	Z	*p*
Smoking	−0.63	−0.03	−0.07	−2.16	0.03
Physical activity	−0.25	0.22	−0.005	−0.15	0.9
Urban and Non-urban	−0.12	0.3	0.03	0.86	0.4
Household income	−0.15	0.25	0.02	0.46	0.7
Age	−0.25	−0.06	−0.11	−3.05	0.002
Educational level	0.08	0.2	0.18	4.76	< 0.001
Mother’s educational level	−0.04	0.01	−0.04	−1.08	0.3
Stress	−0.06	0.02	−0.03	−0.93	0.4

The study also includes c-reactive protein (CRP) data from IFLS waves 4 and 5, with 224 subjects in IFLS waves 4 and 226 in IFLS waves 5 having CRP results. We conducted a separate analysis for CRP, as combining it with other independent variables significantly reduced the total number of subjects. In wave 4 of IFLS, two subjects and in wave 5, four subjects had CRP levels exceeding 15 mg/L (see Table 3). Our study yielded an R2 of 0.67.

**Table 3:** CRP level.

CRP level	IFLS wave 4	IFLS wave 5
High	2	6
Normal	222	220
Total	224	226
Spearman Rho	0.02	0.05

Table 4 showed that there were no multicollinearity, all VIF score below 5 and no Tolerance between dependent variables, all Tolerance score > 0.1.

**Table 4:** VIF and tolerance test.

Variables	VIF	Tolerance
Educational level*	2.89	0.346
Mother’s educational level*	1.88	0.531
Smoking*	1.78	0.562
Physical activity*	1.02	0.978
Stress*	1.04	0.966
Urban and Non-urban*	2.15	0.465
Household income*	1.06	0.939
Age*	4.15	0.241
Educational level **	2.92	0.342
Mother’s educational level**	1.92	0.520
Smoking**	1.51	0.660
Physical activity **	1.03	0.975
Stress**	1.08	0.926
Urban and Non-urban**	2.22	0.451
Household income **	1.10	0.911
Age**	4.07	0.245

*IFLS wave 5.

**IFLS wave 4.

## 3 Discussion

The cognitive function abilities of a child are influenced by several factors, including memory, motor skills, sensory skills, attention, and processing speed (20). The presence of a disorder in any of these factors can cause disruptions in a child’s cognitive functions (5). The cognitive function of a child is important, especially for the achievement of academic success (6, 7). Given the critical role of working memory in overall memory function, as well as the important role of memory function in a child’s cognitive abilities, this forms the background for why this study was conducted.

There are several types of tests commonly used to measure the working memory capacity in children, including the digit span backward test, digit span forward test, free recall test, and N-back test. The free recall test is a test where a person is asked to repeat a number of words (nouns) that have been mentioned before. The free recall test is considered better in reflecting someone’s working memory capacity compared to the digit span test, where a person only mentions a sequence of numbers or letters (5, 20).

Working memory is the capacity to store and process brief or uncomplicated sensory inputs within a limited or specific timeframe (5, 20). It is an indispensable element in the development of cognitive functions (6, 7). It plays a critical part in a child’s learning activities within a classroom setting (8), including the capability to memorize, follow instructions, and solve problems (9, 10). Working memory plays a vital role in mathematics, requiring individuals to mentally remember numbers and their combinations (8). In language abilities, working memory is instrumental in reading, using appropriate language, and formulating sentences that reflect one’s thoughts (11, 12). Therefore, it can be concluded that working memory is essential in supporting a child’s academic performance (21, 22).

This study is the first to identify the determinants of adolescent’s working memory using longitudinal data. By cross-analyzing data extracted longitudinally, separated over the course of 7 years, several determinants that imply causation toward the adolescent’s working memory were identified. Robust subject eligibility ensured reliable results. Many previous studies have been conducted cross-sectionally, highlighting the need for greater consistency in determining factors.

Memory is one crucial fundamental aspect of the construction of cognition. Memory, sensory, motor, inhibition control, processing speed, and attention are critical aspects for adolescents in learning. Working memory is an essential part of memory (23).

This study found a positive association between adolescent’s working memory and household income with educational level. However, there was no association with the mother’s educational level. From our hypothesis, the government’s program on *Wajib Belajar 9 Tahun* (9 years of mandatory schooling) implemented in 1994 may have contributed to this phenomenon (24). The program aimed to ensure every child completes at least 9 years of school or at least junior high school for free (24). To comply with the President’s mandate, the government constructed numerous elementary and junior high schools in all districts and sub-districts throughout Indonesia. This policy enabled every child in Indonesia, regardless of their family’s financial status, to attend and complete their junior high education free of charge. The 9-year compulsory education law applied to all children, even those whose parents opposed education (24). This 9 years of mandatory schooling program was also free, so this explain why poverty was not had any association with education level in this study.

Many parents have various reasons for not valuing education, such as encouraging their children to work to support the family or to marry at a young age. Nonetheless, *Wajib Belajar 9 Tahun* mandates school attendance for all children in Indonesia. In our study, household income and maternal education did not correlate with children’s educational attainment or working memory. However, individuals can improve their working memory through training, and school is one of the ways to do so (25, 26). Working memory is a crucial cognitive function that plays a significant role in classroom activities and academic achievement. Participating in educational activities during classroom attendance can improve pre-adolescent children’s cognitive health and short-term memory performance. Optimizing the effectiveness of the fronto-parietal network in supporting working memory requires stimulating children through educational activities. Social interaction during elementary school has an impact on children’s brain development. Based on research conducted by Kamijo et al., the impact of social interaction on elementary school children is comparably less than that of adults. As a result, brain activity can be enhanced for individuals with a higher level of education (25).

Smoking was found to negatively affect children’s working memory in this study, which aligns with previous research. Pre-frontal cortex was identified as one of the most important area for working memory (27, 28). Smoking children showed not only a smaller prefrontal cortex but also less volume across temporal, parietal and frontal compared to non-smoking children (27). Other study stated, during adolescent when prefrontal cortex has not completed its maturation, exposure of nicotine will be more vulnerable and will affect the working memory process (28). In this study, smoking prevalence was quiet high. Data in IFLS wave 4, when subject age were 15–18 years old, total children who were smoke were 12.6%, which 5% of the start to smoke below 10 years old.

Previous research has consistently demonstrated significant improvement in working memory for adolescents aged 15–18, with a subsequent decline around ages 23–25. Previous research has shown that short-term memory development typically occurs between the ages of 4 and 6 and progressively increases until adolescence, specifically between the ages of 15 and 18 (21). Memory maturation is believed to occur during the middle years of childhood and is followed by a plateau phase, with declines typically observed in later adulthood and old age. This could be attributed to the developmental changes in the prefrontal cortex. Disruptions in the prefrontal cortex can impair attention, memory-related control functions, and organization (4).

The study’s unique methodology of cross-lagged analysis from longitudinal data strengthens associations between variables and identifies causality, providing more convincing results than previous research. Secondly, the inclusion of a substantial sample size consisting of 894 adolescents increases the reliability and strength of the study.

Nevertheless, this research has limitations since it only considers certain variables identified as determinants of working memory in adolescents in previous studies. Therefore, the researchers recommend conducting additional studies that include additional variables to gain a more comprehensive understanding of the factors that influence working memory in adolescents.

## Data availability statement

The original contributions presented in the study are included in the article/supplementary material, further inquiries can be directed to the corresponding author.

## Ethics statement

The IFLS survey and its procedures were approved by review boards in the US (at the Rand Corporation in Santa Monica, California) and in Indonesia (at Universitas Gadjah Mada in Yogyakarta and Universitas Indonesia in Jakarta). Every participant gave written informed consent to participate. For children in the study, this consent was also obtained from their family members, caregivers, or legal guardians.

## Author contributions

HN: Conceptualization, Investigation, Writing – original draft. HS: Conceptualization, Supervision, Writing – review & editing. HH: Validation, Writing – review & editing. MH: Data curation, Formal analysis, Writing – review & editing. AP: Validation, Writing – review & editing.
